# Supplementary material for: Oviposition Preference and Developmental Performance of Drosophila suzukii on Different Cherry Cultivars
Source: Insects. 2024 Dec 11;15(12):984. doi: 10.3390/insects15120984 (PMC11679544; doi:10.3390/insects15120984)
Supplement: Supplementary file 1 [file insects-15-00984-s001.zip › insects-3348821-supplementary.pdf]

Table S1 Ripeness stage classification for different cherry cultivars

Cultivar	Ripe stage	Overripe stage
Hongdeng (HD)	5/16	5/23, 5/29
Tieton (TT)	5/16	5/23, 5/29
Burlat (BLT)	5/16	5/23, 5/29
Xiangquan No.1 (XQ1)	5/16, 5/23	5/29, 6/6
Reinier (RN)	5/23, 5/29	6/6, 6/12
Caihong (CH)	5/16, 5/23	5/29, 6/6

Note: The collection dates in the table are presented in the “month/day” format.

Table S2 Generalized Linear Model (GLM) analysis of cherry fruit cultivars, ripeness stages, and fruit physiological parameters

Response variable	Fixed effect	F	Df	*P* value
*L**	Cultivar	333.01	5	<0.001
	Stage	184.35	1	<0.001
	Cultivar×Stage	20.61	5	<0.001
*a**	Cultivar	88.71	5	<0.001
	Stage	128.71	1	<0.001
	Cultivar×Stage	53.22	5	<0.001
*b**	Cultivar	349.36	5	<0.001
	Stage	678.03	1	<0.001
	Cultivar×Stage	170.04	5	<0.001
CIRG	Cultivar	207.16	5	<0.001
	Stage	218.52	1	<0.001
	Cultivar×Stage	59.12	5	<0.001
Firmness	Cultivar	88.75	5	<0.001
	Stage	404.25	1	<0.001
	Cultivar×Stage	65.5	5	<0.001
Sugar content	Cultivar	140.19	5	<0.001
	Stage	104.84	1	<0.001
	Cultivar×Stage	20.12	5	<0.001

Note: The first four parameters are all related to fruit color. *L**: lightness, *a**: the red–green axis, *b**: the yellow–blue axis, *CIRG*: the color index of red grapes.

Table S3 Physiological data of different cherry cultivars at different ripeness stages

Cultivar	Ripeness stage	*L**	*a**	*b**	*CIRG*	Firmness (kg/cm^2)	Sugar content (°Brix)
HD	R	41.85±1.088	39.33±0.871	35.76±0.506	1.91±0.038	2.51±0.092	18.23±0.261
	O	27.96±0.323	16.91±0.923	5.21±0.440	4.11±0.116	1.78±0.069	21.74±0.357
TT	R	45.64±1.732	24.74±1.554	20.96±0.863	2.30±0.058	6.22±0.192	16.08±0.219
	O	29.77±0.403	22.89±0.936	8.52±0.523	3.45±0.096	2.43±0.057	16.59±0.153
XQ1	R	37.28±1.717	24.58±1.114	16.76±0.856	3.06±0.201	3.01±0.071	15.72±0.169
	O	32.97±0.674	25.06±0.835	10.39±0.607	3.08±0.076	1.80±0.068	15.26±0.245
CH	R	50.06±0.905	26.15±0.782	21.89±0.547	2.13±0.023	4.32±0.209	17.81±0.150
	O	43.15±0.657	26.61±0.574	18.06±0.368	2.39±0.026	2.28±0.067	19.55±0.255
BLT	R	30.51±0.440	24.37±0.826	8.92±0.482	3.22±0.067	1.86±0.136	17.94±0.248
	O	25.54±0.249	6.95±0.666	1.05±0.298	5.66±0.102	1.65±0.076	22.29±0.442
RN	R	57.58±0.628	15.72±0.803	24.88±0.411	2.05±0.017	2.35±0.114	15.80±0.165
	O	57.06±0.684	12.64±0.803	26.24±0.508	2.07±0.020	2.18±0.106	17.06±0.255

Note: In the "Ripeness stage" column, "R" represents "Ripe stage" and "O" represents "Overripe stage". *L**: lightness, *a**: the red–green axis, *b**: the yellow–blue axis, *CIRG*: the color index of red grapes. All data are presented as average values ± standard errors (SDs).

Table S4 Results of t-test comparing differences in fruit physiological parameters between ripeness stages

Parameters	Cultivar	t	df	*p* value
*L**	TT	8.92	32.171	<0.001
	XQ1	2.33	76.749	0.022
	HD	12.24	34.205	<0.001
	BLT	9.83	48.15	<0.001
	CH	6.18	107.68	<0.001
	RN	0.56	117.13	0.574
*a**	TT	1.02	50.593	0.3146
	XQ1	-0.34	109.35	0.735
	HD	17.66	80.675	<0.001
	BLT	16.41	65.357	<0.001
	CH	-0.47	108.29	0.634
	RN	2.71	118	0.008
*b**	TT	12.32	50.87	<0.001
	XQ1	6.06	106.39	<0.001
	HD	45.6	69.825	<0.001
	BLT	13.88	51.677	<0.001
	CH	5.82	103.33	<0.001
	RN	-2.08	113.13	0.04
CIRG	TT	-10.28	86.279	<0.001
	XQ1	-0.09	75.723	0.928
	HD	-18.16	70.564	<0.001
	BLT	-20.07	87.684	<0.001
	CH	-7.54	116.36	<0.001
	RN	-0.78	114.01	0.434
Firmness	TT	18.94	34.283	<0.001
	XQ1	12.25	117.73	<0.001
	HD	6.37	61.748	<0.001
	BLT	1.33	47.448	0.189
	CH	9.27	70.918	<0.001
	RN	1.04	115.9	0.302
Sugar content	TT	-1.94	57.426	0.057
	XQ1	1.57	104.92	0.119
	HD	-7.94	87.863	<0.001
	BLT	-8.58	84.872	<0.001
	CH	-5.88	95.355	<0.001
	RN	-4.15	101.09	<0.001

Note: *L**: lightness, *a**: the red–green axis, *b**: the yellow–blue axis, *CIRG*: the color index of red grapes. All data are presented as average values ± standard errors (SDs).

Table S5 Non-choice oviposition of *Drosophila suzukii* on different cherry cultivars and ripeness stages, and t-test analysis results

Cultivar	Ripeness stage	Average egg amount	t	df	*p* value
HD	R	32.0±4.8	0.024	9.65	0.981
	O	31.9±2.0			
TT	R	13.4±2.9	-2.2861	11.425	0.042
	O	20.9±1.6			
XQ1	R	12.4±1.4	-3.0107	21.864	0.006
	O	21.6±2.8			
CH	R	4.1±0.6	-5.634	21.911	<0.001
	O	12.1±1.3			
BLT	R	23.5±4.4	-1.63	9.7	0.135
	O	31.3±1.9			
RN	R	8.8±1.2	-3.252	25.44	0.003
	O	15.9±1.8			

Note: In the “Ripeness stage” column, “R” represents “Ripe stage” and “O” represents “Overripe stage”. Average egg amount is presented as average values ± standard errors (SDs).
